# Building on our momentum

**DOI:** 10.1002/jsp2.1226

**Published:** 2022-09-29

**Authors:** Robert Mauck, Daisuke Sakai, Mauro Alini

**Affiliations:** ^1^ University of Pennsylvania, McKay Orthopaedic Research Laboratory Philadelphia Pennsylvania USA; ^2^ Orthopaedic Surgery Tokai University School of Medicine Graduate School of Medicine Isehara Japan; ^3^ AO Foundation, AO Research Institute Davos Switzerland

Dear Readers,

As noted in the last issue, we were overjoyed that the *JOR Spine* received its first impact factor (IF)‐3.7 in 2022! While only one part of what distinguishes the *JOR Spine*, this underscores the outstanding content and mission of the journal. Now that we have achieved this first milestone, we turn our attention to continuing our goal of becoming the top journal within the spine basic and translational research field.

The continued strength and growth of the Journal depend on many factors, most important of which is you, our readers and contributors. The editorial team, in consultation with the Advisory Review Board, are working hard to program new content that further enriches the Journal and expands its reach and impact. We also look forward to seeing many of you and getting your feedback (and celebrating) at this year's International Philadelphia Spine Research Symposium (6–10 November; https://www.ors.org/event/2022-ors-psrs/) where we will provide an update on the remaining milestones for *JOR Spine* and our plans to build on this momentum.

We would also like to bring to your attention the open call for the 2023 *JOR Spine Early Career Award*. This award, developed in collaboration with the ORS Spine Section, is designed to recognize the “rising stars” of our field and highlight their outstanding work published in the *JOR Spine*. For each of the past 3 years, awardees have been recognized at the ORS Annual Meeting, and we look forward to continuing this tradition in the coming year. Applications for this highly competitive award are due 31 October 2022 and information on the award nomination process and eligibility criteria can be found at: https://onlinelibrary.wiley.com/page/journal/25721143/homepage/earlycareeraward. Please consider nominating your colleagues (or yourself) for this Award!

As we move forward, in a new world of in‐person interactions, we look forward to seeing and working with all of you to build on the momentum of JOR Spine, and to make it the go‐to resource for the international spine research community.

See you all soon!

Rob, Dai, and Mauro.

